# “It’s all about time and timing”: nursing staffs’ experiences with an agile development process, from its initial requirements to the deployment of its outcome of ICT solutions to support discharge planning

**DOI:** 10.1186/s12911-022-01932-4

**Published:** 2022-07-17

**Authors:** Sofi Nordmark, Inger Lindberg, Karin Zingmark

**Affiliations:** 1 Department of Research and Education, Region Norrbotten, Luleå, Sweden; 2 Division of Nursing, Department of Health, Education and Technology, Luleå University of Technology, Luleå, Sweden; 3 Division of Nursing, Umeå University, Umeå, Sweden

**Keywords:** Collaboration, Agile, ICT, Experiences, Nurses, Homecare organizers, Discharge planning, Qualitative content analysis, Descriptive statistics

## Abstract

**Background:**

Agile projects are statistically more likely to succeed then waterfall projects**.** The overall aim of this study was to explore the nursing staffs’ experiences with an agile development process, from its initial requirements to the deployment of its outcome of ICT solutions aimed at supporting discharge planning.

**Methods:**

An explorative design with quantitative and qualitative methods was used. Qualitative data was collected through seven focus group interviews. Quantitative data was collected via an ICT-system, and with an evaluation form submitted by fourteen registered nurses and nine district nurses.

**Results:**

Qualitative result of the experiences with the agile development process and its outcome resulted in one theme, four categories, and ten subcategories. The theme was found to be about time and timing, namely the amount of time for the different activities and the timing of activities within and between organisations. The agile development process increased the participants’ readiness for change by offering time to learn, practice, engage and reflect, and then adopt the ICT as a support to daily practice. Quantitative results showed a variated adoption of the ICT.

**Conclusion:**

There is a need for time to prepare, understand and adopt new tools, services and procedures and a need for additional time to prepare, understand and adopt the new among individuals, collectives, organizations, and sometimes even between different collectives or organizations. The agile development process offered the end-users involvement through the development process, which gave them time to change it both individually and collectively. However, there is a need for close collaboration between the development project team and management to reach an organizational change that is timely for both the individual and the collective change. When time or timing fails in the development or implementation process, there is a huge risk of non-adoption of new tools, services, or procedures or among the end-users.

## Background

All of society is involved in the massive movement towards digitalization, and healthcare is no exception. Information and communication technology (ICT) includes all digital technology that facilitates the electronic capture, processing, storage and exchange of information [1]. Examples of ICT used in healthcare include electronic medical records, medical decision support, computer-aided learning, telecare and medical imaging [2]. However, not all digital solutions have led to better health outcomes and values. Persson and Rydenfäldt [3] point out that many digital healthcare systems are poorly designed. They force users into inefficient workflows where workarounds become permanent solutions. Deficits like the lack of interoperability between the digital systems and limited support for cross-organizational teamwork causing an increased flow of information are common [4]. Besides technological factors that influence the success or failure of ICT implementation, human and organizational factors, such as knowledge and attitudes, work culture, staff stability/shortage and leadership, are also crucial [5].

Every day people are discharged from hospital, some fully recovered while others need home help and support. Collaboration and timely information exchange between care providers are essential for a safe and secure care transition. Poorly managed care transitions have been directly linked to adverse events, readmissions, increased costs and death [6–8]. In order to facilitate and improve the discharge process and to prevent undesirable outcomes, different interventions involving ICT solutions have been developed over the years. Different decision-supportive tools [9, 10], tailormade discharge models and structured discharge summaries [11–13] have been tested with various results. A review of statistics published between 1999 and 2016 show that 7–89% of the ICT projects in different sectors failed because they did not meet one or more of three basic criteria: finishing on time, finishing within budget, and achieving satisfactory results [14]. The schedule overrun varied between 7 and 87%, the cost overrun varied between 16 and 89%, and failure to reach expected values or project requirements varied between 7 and 75%. Also 15% of software projects never delivered anything, 5–31% were cancelled before completion and 15–28% were cancelled before implementation. The success rate varied between 12 and 34%. On the other hand, measuring software project success based not only on the three basic criteria, but also on product success, business success and strategic success of the organisation, increases the success rate up to 70%. This raises the questions: do we understand the complexity of these tools, services, and procedures and are we measuring the correct things?

There are different methodologies and models for ICT development projects. The best fit depends on the project’s aim and its limitations. The literature describes different project management approaches, including traditional ones (e.g., waterfall methodologies), agile methodologies and hybrids, which are a combination of traditional and agile [15]. Wysocki [16] describes traditional, agile, and extreme approaches and distinguishes them by their life cycles, i.e., as being linear, incremental, iterative, adaptive, or extreme models. The waterfall approach is a linear, sequential software development process wherein the progress flows toward the conclusion through separate phases in which the activities are executed one by one [17]. The following phases are required: data collection, analysis, design, development, and testing. If successful, these phases will be followed by implementation and maintenance. It focuses on an extended documentation and planning stage before its actual creation. Once the project begins, the waterfall methodologies resist changes. The Agile approach, on the other hand, is built on four values and twelve principles, stated in the Agile Manifesto, and focuses more on people, results, collaboration and flexible responses to change [18]. The agile software development process is an iterative and adaptive process, where the progress is separated into small sprints that allows for necessary changes to be made at any time since testing is performed concurrently with development. The dialogue and collaboration between the customers/users and developers are the backbone of this process. According to Alsaqqa et al. [19] agile methods are processes that support the agile principles and describe how the day-to-day work is performed by the software developer. There are a variety of agile methods that differs from the other by the set of terminology and practices, including Scrum, the dynamic systems development method (DSDM), feature-driven development (FDD) and lean software development. However, a uniform definition for the concept of agile methods has not been established. According to Mnkandla and Owolatzky [20] Scrum, DSDM and FDD can be defined as agile methodologies that follow the values and principles in the Agile Manifesto. There are advantages and disadvantages to both waterfall and agile methodologies. The waterfall is easy to understand, systematic, and well documented [21]. It is suitable for small projects where the influencing factors are well known and the requirements are well defined. Since the methodology allows neither changes nor going back to the previous phase during the process, it is not suitable for larger projects or situations where accurate requirements and needs are difficult to identify and document. Testing only at the end of the development project carries a huge risk of failure or lack of quality in the end product. The agile methodology, on the other hand, is suitable for projects where the requirements are not obvious or where even the goal is difficult to define from the outset [22]. This methodology helps the customer to define their needs and requirements through close collaboration with the developers. Small deliveries (sprints) every two to four weeks throughout the whole project promote early discovery of failures and thereby opportunities to make necessary changes throughout the whole process. Sharma, Sarkar and Gupta [23] state that the advantages of the agile methodology can also become disadvantages. If customers do not communicate their desires clearly to the developers, ambiguity can result creating delays and higher costs. A change of team members can also mean a loss of knowledge since the methodology does not focus on comprehensive documentation, in contrast to the waterfall methodology. Nevertheless, agile projects are statistically more likely to succeed than waterfall projects [24]. Depending on the criteria used and how they are measured, studies indicate that agile projects are 12–73% more successful than waterfall projects.

The digital transformation of healthcare includes the need to adapt current routines, workflows and organisational cultures as part of change management [25]. Using agile methodology in the public sector comes with several challenges. Organizational culture is often very hierarchical, with inflexible rules, regulations and politics and a lack of involvement and participation of the project’s end user [26]. A 2019 literature review of the use of agile methodologies in implementing ICT within healthcare indicated that there is very little literature available [27]. However, the COVID–19 pandemic seems to have forced the healthcare sector to embrace agile development processes to be able to rapidly handle the uncertainty and needs created by the pandemic [28]. Under pandemic conditions, goals and requirements often changed by the time a model was deployed, thus demonstrating that the waterfall methodology was unsuitable. However, Lim et al. [29] managed to develop a functional COVID-19 symptom monitoring system within nineteen days using an agile methodology. The main challenges were time factors and communication gaps between the technical and clinical teams. On the other hand, clearly defined and communicated roles meant that everyone knew who was doing what, when, and this became a strength of the project, while also saving time. Another timesaving feature was having enough team members to allow roles and tasks to be covered if someone was not available [30]. Cheung et al. [31] describe a similar six-week development process of a care model for children with an inflammatory multisystem syndrome associated with SARS-CoV-2. They emphasize the importance of defining a starting point from which improvements can be made by prioritising data collection throughout the process along with an early implementation. At an early stage, they identified relevant team members and an early communication strategy in order to eliminate the risk of failures and delays. A fragmented decision-making process and randomly communicating outcomes gave the appearance of speed but often led to delayed decisions. Communication and cooperation challenges are common in the development process of an eHealth project, especially where experts from different organisational cultures and backgrounds are involved [32]. Preventing communication failures and providing personnel with the necessary information and knowledge will prepare the client for change and enable them to contribute to the transformation process [25].

There is a clear need for research to explore project management methodologies and identify weak areas in the project life cycle in order to meet the needs of increased ICT adoption. There is a consensus among policymakers and researchers that problems with ICT projects arise from sociological, cultural, and financial issues [33]. According to the literature, ICT implementations in healthcare continue to be a challenge and would benefit greatly from further research on how agile methodologies have been used for IT projects within the healthcare setting, including an understanding of project outcomes. There is also a need consider that measurements of project success have evolved from the original aspects of time, cost and scope to include customer satisfaction and benefits to stakeholders. However, project success is defined differently by various project management approaches. Combining qualitative and quantitative evaluations of agile project management used in ICT projects in healthcare settings could help reduce the high percentage of ICT project failures.

## Methods

### Aim

The aim of this study was to explore nursing staffs’ experiences with an agile development process, from its initial requirements to the deployment of its outcome of ICT solutions aimed at supporting patient discharge planning.

### The development project

This study was part of a large development project called Future Innovative Work Practices in Health Care and Social Care (FIA), which was conducted in northern Sweden [34]. The project aimed to develop work methods and ICT solutions that increased the accessibility, safety, quality, and efficiency of healthcare, while also creating regional growth and lowering costs. The project met the three basic criteria of finishing on time, finishing within budget and having satisfactory results. For patient discharge planning (DP) in this county, the *Region Norrbotten* and all municipalities adhere to a 2001 agreement that all patient-related information exchanges will occur using a common electronic information exchange system. The IT system (a web application) had all data stored on a central server located at an IT company that operated the system. The IT system was integrated with the Swedish population register and the *Region Norrbottens* electronic health record system. As a minimum, information concerning discharge planning should be exchanged when the patient is admitted to the hospital, when a discharge planning conference (DPC) is requested, during the DPC and when the patient is discharged. The information exchange was routinely performed between registered nurses (RNs) at the hospitals, district nurses (DNs) at the healthcare centres, and healthcare organizers (HCOs) within the municipalities. If necessary, other professions could also participate in the DP.

Using statistical data regarding DPs performed over the past two years between a healthcare centre involved in the project application and the central county hospital, invitations to participate in the project were extended to the five hospital wards with the highest frequency of DPs: geriatric/palliative, infection, surgical, orthopaedic, and medical. Two additional healthcare centres were then invited due to their geographic locations. One healthcare centre was located in a smaller town with patients spread over a large geographical area, a second was in a larger city close to the hospital and the third was in the countryside. Two municipalities that served patients in the same care catchment area were also invited to participate in the project.

The development project followed the principles of the Agile Manifesto [18], which emphasizes that close collaboration and regular meetings between customers and developers throughout the process is essential. The first author had access to all the project documentation stored at the e-Health Innovation Centre. The project contained the following components (also presented in Table 1):

- Project planning, project application and establishment of the project organisation;
- Information meetings at each workplace for all staff and management about the project’s aim, schedule, and estimated resources;
- Three workshops mapping the current and the desirable DPs, including subprocesses, activities and actors, to identify and specify problem areas and requirements;
- Iteration of project meetings every three weeks to design, develop and test supportive ICT solutions suitable for the needs of RNs, DNs and HCOs and their daily work with DPs;
- Training in the new ICT solutions and new routine operations;
- Testing of new ICT solutions in a twelve-month pilot system (The staff involved were offered support via phone or email 24/7 during the test period. A printed quick reference guide, was also available at every setting.);
- Project meetings every second month for follow-up and the exchange of information and opinions during the pilot;
- Evaluation of the development process and new ICT solutions at the end of the twelve-month pilot.
- Information meetings after the pilot to communicate the results and allow senior management to decide on scaling up or closing down the project;
- Evaluation of the ICT solutions in use after one year; and
- Evaluation of the ICT solutions after five years.

**Table 1:** Description of participants at each main project activity

	Information meetings before development begins	Workshops	Project meetings and design, development and tests	Internal project meetings	Training	Pilot	Project meetings during pilot	Focus group interviews	Information meetings after pilot
Twelve participating RNs (hospital)	x	x	x		x	x	x	x	x
Nine participating DNs (healthcare centre)	x	x	x		x	x	x	x	x
Four participating HCOs (municipality)	x	x	x		x	x	x	x	x
All involved RNs, DNs and HCO at five hospital wards, three healthcare centres and two municipalities	x				x	x			x
Patients and relatives involved in videoconference						x			
Unit/middle management involved (region and municipalities)	x	x				x			x
Division/senior management (region and municipalities)	x								x
Project manager (university)	x			x					x
Internal technology project leader (region)	x		x	x			x		
Scrum master (university)	x	x	x	x	x	x	x		x
External technology project leader (two external companies)			x	x					
Internal technology developers (regional level)			x	x					
External technology developers (two external companies)			x	x					
Internal help desk (regional level)					x	x			x

The agile development process led to the following five ICT solutions aimed at supporting the DP and which were also tested in the pilot:

1. The Polycom CMA Desktop videoconferencing system was tested as a new way for the DPC to meet. A Logitech Webcam C910 HD with a laptop, desktop or 42-inch flat screen was used to view the video imaging. Equipment used to send and receive signals included an external microphone, a Logitech USB Desktop Microphone and external speakers, Logitech S-120, or a headset, a Logitech Clear Chat comfort USB. Additionally, a new combined microphone/speaker with sound reduction, the Phoenix Audio Technologies Duet PCS, was tested.
2. A shared electronic web calendar that can be accessed and used by RNs, DNs and HCOs together to synchronize times for DPC meetings was developed and tested.
3. In collaboration with the IT company operating and maintaining the electronic information system used for information exchange at the DP, the need to replace unstructured free-text data was recognized and attached files with structured information about the status of the patient’s active daily living (ADL) that can be sent to DNs and HCOs with a request for a DPC were developed and tested.
4. A common form for outpatient follow-up about the agreed discharge plan in the existing electronic information system that can be shared and updated by DNs and HCOs on different occasions while remaining available for RNs, DNs and HCOs to read was developed and tested.
5. A surveillance list in the existing electronic information system to keep track of outpatients who need follow-up of their discharge plan after hospital discharge was developed and tested.

### Design

The development project was designed in accordance with the principles of the Agile Manifesto [18], while the study was based on the new Medical Research Council (MRC) guidance for developing and evaluating complex interventions [35]. An exploratory design with a qualitative approach was chosen to capture RNs’, DNs’ and HCOs’ experiences with the agile development process, from initial requirements to the deployment of the ICT solutions aimed at supporting the DP. In line with the MRC Framework [35] both qualitative and quantitative data were used. All methods were carried out in accordance with relevant laws, guidelines, and regulations. Qualitative data were collected through focus group interviews (FGs) [36] to obtain data about the experiences of the agile development process and testing of all five ICT solutions. FGs can produce rich insights into realities defined in a group context and expressed by beliefs, perceptions and views. Most of the focus was on the videoconference because it engaged the patients and their relatives, whereas the other four ICT solutions were administrative tasks involving different caregivers. Because no acceptance testing of the technology was performed, quantitative data were collected using an evaluation form [37] to obtain a more specific view of how the videoconference technology was working at an early stage of the pilot. Quantitative data about usage frequency were also collected from the old and new electronic information systems. The old system was in use during the pilot and at the first follow-up one year later. However, it had been replaced with a new one by the time of the second follow-up five years after the pilot had taken place.

### Participants and procedures

#### Participants in the focus group interviews

Twelve RNs from five hospital wards, nine DNs working with in-home-nursing patients at three healthcare centres and four HCOs from two municipalities were asked to participate in the FGs. All but one RN agreed to participate. Seven FGs were performed with twenty-four participants (as presented in Table 2). In Focus Group 3, one informant had to leave after a few minutes due to an urgent patient case, but the other two wanted to continue.

**Table 2:** Description of participants and focus groups

	FG1 Primary healthcare centre 1	FG2 Primary healthcare centre 2	FG3 Primary healthcare centre 3	FG4 Hospital wards 1 and 2	FG5 Hospital wards 3 and 4	FG6 Hospital ward 5	FG7 Municipalities 1 and 2
Informants	3 DNs	3 DNs	3 (2) DNs	2 + 2 RNs	2 + 2 RNs	3 RNs	2 + 2 HCOs
Age, minimum–maximum/average	38–59/50.3	39–58/46.6	50–53/51.6	39–42/40	39–45/42.7	36–50/41	45–58/51.2
Professional work experience, min.–max./average							
As an RN	2–18/8	4–7/5.6	5–16/9.3	8–17/13.6	2–22/13	8–17/12.6	
As a DN	4–21/14	2–13/9	4–26/13.3				
As an HCO							1–12/7

#### Participants in the evaluation study

Fourteen RNs at five hospital wards and nine DNs at three healthcare centres working with home-nursing patients and performing DPC through videoconferencing were asked to complete the evaluation form. All agreed to participate.

### Data collection and analysis

#### Focus group interviews

At the end of the pilot, data about the agile development process and the ICT solutions under study were collected through FGs. The primary author was the moderator during all the FGs, and a senior supervisor was also present to take notes and ask clarifying questions, as needed [36]. Both researchers had experience with conducting FGs. The moderator was well known among the participants due to the development stages of the project and also a former role as an IT administrative manager at Region Norrbotten. During the FGs, vignettes related to the developed and tested supporting ICT tools were shown. A semi-structured interview guide was developed using the results of the quantitative data and other resources. Questions were asked such as “How much have you been involved in the development and testing of X?” and “How has it worked? Tell me about obstacles and feasibilities you have experienced?” Additional questions were also asked such as “Did you receive training in the ICT tools?” and “Have you received support if needed?” The length of the FGs varied between forty-one and seventy-four minutes, and the average time was sixty-five minutes. The focus group interviews took place in a room outside their workplace and were recorded and transcribed by the primary author. The interviews were analysed using qualitative content analysis to describe the manifest and latent content of the text [38]. The interviews were read several times to obtain a sense of the whole before meaning units corresponding to the study’s aim were extracted from the text. Microsoft Excel (version 16.0) was used to support the organizing of the meaning units into areas of the agile development process and the developed ICT solutions. The meaning units were condensed with regard to the content and then coded. The various codes were compared to identify similarities and differences before codes with similar content were grouped together into subcategories. The categorizing was performed in several steps to form broader categories by abstraction, resulting in four categories. The categories were compared by moving back and forth between text units, subcategories and categories to identify patterns from which one theme was interpreted and formulated. Each step of the analysis was discussed by all three authors.

#### Evaluation form

To achieve an early evaluation of whether the RNs, DNs and HCOs had experienced the videoconferencing technology to be reliable in this context, data about the videoconferences were collected over a three-month period through an evaluation form at the beginning of the pilot. The evaluation form was originally designed and used to evaluate distance consultation by videoconferencing in another study [39]. The form was modified by the primary author and the technical project leader to suit the study at hand (i.e., the new context) after gaining permission from the original designer. Questions about the reason for establishing contact, the supervision and the outcomes of consultation were deleted. The evaluation form contained six questions about sound and picture quality as well as one question about the experience of conducting videoconference meetings. All the questions were scored on a four-point Likert scale from very pleased to very displeased. RNs and DNs were asked to complete the form after conducting a videoconference. Data from the evaluation form were analysed using descriptive statistics [37] with IBM SPSS Statistics 21.0.

#### Electronic information system

Data were extracted from the electronic information system aimed at facilitating the exchange of information among hospitals, healthcare centres and community homecare providers that were in use during the pilot and from the new system in place during the five-year follow-up. Data were analysed using descriptive statistics [37] and Microsoft Excel (version 16.0).

## Results

The result is presented under these three headings based on the agile-inspired development process life cycle (planning and analysis, iteration of requirements, design and development delivered in sprints, piloting, deployment, and maintenance).

- Qualitative results of the nursing staff’s experience with the agile development process from requirements to piloting the ICT solutions, as collected in the focus group interviews.
- Qualitative and quantitative results of the technology’s use and the nursing staff’s perceived satisfaction during the piloting of the developed ICT solutions.
- Deployment and maintenance: scaling up or closing down

### Qualitative results of providers’ experiences with the agile development process, from requirements to piloting of ICT solutions, as expressed in focus-group interviews

The qualitative analysis explored the agile development process concerning the following interventions: videoconferences and related equipment, the development and testing of a shared electronic web calendar, attached files with information about the patient’s ADL status, a common form for outpatient follow-up of the agreed-upon discharge plan, and a surveillance list to keep track of outpatients in need of follow-up after hospital discharge. The analysis of the experience with the agile development process from requirements to the piloting of ICT solutions for the DP resulted in one theme, four categories, and ten subcategories*,* as presented in Table 3.

**Table 3:** Interpreted theme with related categories and subcategories

Theme	It’s all about time and timing			
Categories	Allot time for time	Shared time saves time	Gaps in timeliness hinder time and timing	Not if, but when and how
Subcategories	Allocated time for participation creates engagement	Transparency promotes collaboration and planning	The development phase is initially time- consuming and generates excess work	A suitable alternative
	Allocated time for education and practice is essential for improvement and adoption	Transparency increases timely coordination	Consistencies in information and timing between the project and other levels of the organization is necessary	Must be adopted but does not suit all situations
	Timely allocated and initiated support is required			Has received great interest

#### It’s all about time and timing

The agile development process, from initial requirements to piloting ICT solutions aimed at supporting DP, were found to be matters of time and timing. Time was a factor in each of the different activities and for timed activities within and among organisations. The agile development process increased the participants’ readiness for change by offering them time to learn, practice, engage and reflect, resulting in their adoption of the ICT as a support for daily practice. By taking an active part in the development process, they could influence the work and the ICT solutions to become well suited for their daily work with discharge planning. Allowances from management, colleagues, and themselves to allot time on time for involvement and practice was essential for participation in the project.

The participants also became aware of feasibilities and hindrances at the individual, group and management levels that influenced both the development and use of the software. Shared time saves time, and transparency was found to facilitate collaboration, planning and coordination. The timing of training in relation to the initial stages of ICT testing and the timing of support could either facilitate or hinder the work in the development phase. Lack of time and inadequate timing between the project and other levels of the organization were sometimes perceived as a hindrance, e.g., contradictory information and policies preventing timely information exchange. The participants also had to address the fact that the development process was initially time-consuming and required extra work. However, the participants were convinced of the importance of implementing the developed ICT solutions for DP, as reflected in one participant’s phrasing: “not if, but when and how”.

#### Allot time for time

RNs, DNs and HCOs expressed a positive view of the agile development process and of the opportunity to be involved throughout the entire development process. By being involved, they felt engaged and listened to. They stated the importance of allotting time for engagement by themselves, by colleagues and by middle management.

Time for training on and practice with new ICT tools was partially seen as a necessity for the performance of intervention tests and adoption. RNs, DNs and HCOs perceived that they received sufficient education in new interventions and new routine operations. However, they noted that the time between the training and the pilot was so long that their knowledge had lost its effectiveness. Many of them stated that they had forgotten what they had learned before they could apply it. A few of them did not have the time to attend the training sessions and were therefore self-educated with the help of colleagues and a quick reference guide received by all the workplaces before the pilot began. All agreed, however, that no matter how much training they received, the most important factor was allotting time for individual practice in order to become skilled at handling the new technology. However, they experienced both insufficient time and pressure to avoid making mistakes as hindrances to practicing their skills.

It was also important to receive suitable support in a timely manner. During the pilot, RNs and DNs experienced useful and timely telephone support that was also sometimes stressful and time-consuming. They became stressed when they were busy on the phone with support while patients, relatives and other professionals were waiting. They would rather have someone from the IT department provide in-person support and training during the first month. One RN summarised this in the following:

We received enough education, but you need to take time for practice. [...] If you don´t use the new technology, you will forget what you have learned (RN).

#### Shared time saves time

The agile development process meant regular gatherings in which RNs, DNs and HCOs met together and discussed the strengths and weakness of the DP on several different levels. All three different professions perceived that working together across organizational boundaries during the development process produced a transparency that increased their understanding of each other’s roles and responsibilities, which promoted better collaboration and decreased misunderstandings. This was perceived as reducing the time spent on their daily work during the DP, as the communication began to flow more smoothly.

#### Gaps in timeliness hinder time and timing

All three professions expressed frustration with the gap between the middle management’s decision to cooperate in the development project and the providers not receiving the tools to do so. The RNs described one ward not being able to practice videoconferencing more than once due to a lack of a suitable room. After one year, they were still waiting for management to solve the room issue.

Members of all three professions noted that current legislation and routine operations for DPs were communicated unclearly by senior management during the period of the development and pilot phases, which resulted in confusion. They received contradictory messages from senior and middle management concerning the type of patient-related information that was legal to share electronically between the hospital, primary healthcare providers and municipal providers, which impeded ICT testing. The RNs, DNs and HCOs were all very clear that this issue needed to be resolved by the management before implementation could be performed. For example, one RN said,

*The last information we received from the senior management does not agree with our routine operations at all; it has confused many of us so that now we don´t know what information we can document and what we cannot* (RN).

#### Not “if” but “when and how”

By taking an active role in the development process, the RNs, DNs and HCOs perceived they could influence the work and affect the ICT solutions to be well suited for their daily work. This way of developing of new ICT solutions for healthcare was described as demanding and time-consuming but still preferred for future development work. The question is not *if* the personnel should be involved and engaged; it is more *when* and *how*. As one HCO put it:

We need to implement it into our routines and to see the benefits from it [...] not only the excessive workload [...]. Today we just do things around the patient [...] I can see we would obtain a more coherent care process for the patient (HCO).

### Quantitative and qualitative results of the use and perceived satisfaction with the developed ICT solutions during the pilot stage

#### Videoconferencing

A total of forty-seven evaluation forms were returned (RNs n = 37, DNs n = 9), which covers about one-third of the performed videoconferences. The same person could participate in more than one videoconference and then fill in an evaluation form for each conference. Two out of three primary healthcare centres were represented, but only two out of five hospital wards were represented.

In the quantitative analysis, the participants showed an overall high degree of satisfaction with the videoconferencing technology during the DPCs. They expressed high satisfaction regarding image quality, colour, sharpness, and blurring, but they did have some concerns about the sound levels and quality (Table 4).

**Table 4:** Assessment by RNs and DNs of sound and image quality as well as overall satisfaction with DPCs used during videoconferencing

Variables	Very pleased% (n)	Pleased% (n)	Displeased% (n)	Very displeased% (n)	Missing% (n)
Sound quality	30.4 (14)	34.8 (16)	13.0 (6)	19.6 (9)	2.2 (1)
Sound level	32.6 (15)	41.3 (19)	4.3 (2)	19.6 (9)	2.2 (1)
Image quality	43.5 (20)	43.5 (20)	8.6 (4)	2.2 (1)	2.2 (1)
Image colour	41.3 (19)	52.1 (24)	2.2 (1)	2.2 (1)	2.2 (1)
Image sharpness	43.5 (20)	47.8 (22)	4.3 (2)	2.2 (1)	2.2 (1)
Image motion blur	43.5 (20)	50.0 (23)	2.2 (1)	2.2 (1)	2.2 (1)
*Perceived satisfaction with videoconference during DPC*					
DNs	33.3 (3)	55.6 (5)	11.1 (1)	0 (0)	0 (0)
RNs	37.8 (14)	35.2 (13)	18.9 (7)	5.4 (2)	2.7 (1)

An analysis of the qualitative data from the FGs confirmed the problems with the sound at the beginning of the pilot; the participants stated that they could not hear each other. To solve the problem in the moment, they used a telephone with speakers as a supplement to the ongoing videoconference. This problem was addressed by upgrading the CMA client and changing the hospital wards’ headsets to a conference phone with a sound filter. According to RNs and HCOs, many patients had problems with their vision and hearing, which had resulted in difficulties recognizing the DN on a small screen. RNs and HCOs described videoconferencing as working better for older patients if the screen was larger, e.g., a 42-inch screen worked better than a 14-inch one. With a 42-inch screen, several older patients immediately recognized the DN and easily performed the DPC because it reminded them of live television with the DN appearing at near actual size.

DNs who attended the videoconference alone at the healthcare centre perceived difficulties in “taking up space”, and they felt forgotten, especially if there were several people attending the same videoconference meeting at the hospital. DNs thought that if they all became more familiar with the new way of meeting, this issue would automatically be solved:

I feel I became an inactive participant, and it became harder to interact in the discussions [...] It´s probably about practice, to learn the new technology and become more hard-nosed (DN).

They all agreed that the image added value for the patients because it felt more personal to talk with a someone they could see and hear, as compared to a telephone conference. RNs, DNs and HCOs all stated that videoconferencing was not suitable for all patients or in all environments. Some perceived that patients with severe dementia were distracted by the video image, making it difficult to perform the DPC. They would also rather perform a DPC in person with palliative patients. They felt it was more ethical to sit next to these patients in a more personal way and discuss their post-discharge needs.

Due to the long distances DNs, HCOs and patients’ relatives must travel to the hospital, as well as limited personal resources, videoconferencing was seen as a good alternative for the timely completion of the DPC. The design of the videoconference environment was described as either facilitating or hindering the performance of the DPC. In the end, it was stated that the most important factors were the patient’s state of health, individual needs and resources, which should determine the type of meeting arrangement that is most suitable for a timely DPC. RNs and HCOs reported great interest in using videoconferencing and being involved in the development process from caregivers and patients’ relatives. Positive rumours describing the benefits of videoconferencing had some unplanned results. Healthcare centres not involved in the project asked to conduct DPCs via videoconferencing, and some even installed equipment. They all described the system’s economic benefits and environmental savings in addition to the benefits of saving time DNs would otherwise spend traveling to the hospital. RNs, DNs and HCOs had a vision of offering relatives the opportunity to participate in a DPC via videoconferencing in the future. Many elderly people had relatives who lived far away, so this intervention would increase their chances of being involved in the patient’s discharge planning, thereby supporting and empowering the patient. This was expressed in the following:

We have made videoconferencing standard in these three healthcare centres and in some outside the FIA project by their own request (RN).

#### Electronic calendar

The development of a transparent and shared electronic calendar facilitated finding a time to coordinate personal resources across organizational boundaries to ensure timely DPCs. The timely exchange of patient-related information among caregivers was also perceived to be feasible for the DP. RNs, DNs and HCOs perceived that a transparent tool for booking helped them plan dates and times for the performance of DPCs and eased the collaboration necessary for a DPC. RNs reported that in the past they had spent considerable amounts of time coordinating different professionals before a meeting could be scheduled but having access to the other professionals’ schedules/diaries saved time when coordinating DPCs. HCOs expressed only positive thoughts about and experiences about the new shared electronic calendar. They could easily input their available hours, painlessly update changes due to personal resources and save time by not needing to phone the RNs and DNs about changes. One HCO said,

I believe the electronic calendar has worked super well [...]. The big concern is if we end up with nothing [...] that would really be like moving fifty years back in time (HCO).

#### Attached file for ADL

Both RNs and DNs expressed that the new ICT solution of having an attached file with the ADL status of recording patient-related information was supportive but also generated excessive work. RNs and DNs had already documented much of the information in the electronic medical record, to which RNs and DNs (but not HCOs) had access. Double documentation was seen as time-consuming.

#### Form for follow-up of the agreed discharge plan

Only a couple of participants in the FG had tested the follow-up form, so most participants had no or little experience to report. However, they stated that this was partly due to the lack of established routines for how the work should now be done. Both DNs and HCOs thought that the new ICT follow-up form and surveillance list were supportive but labour-intensive due to double documentation in different IT systems.

#### Surveillance list

Since only a few people had tested the follow-up form, the surveillance list had the same result. The participants thought these two interventions were a good way to ensure the patients’ care after their discharge from hospital, but they had not adopted them or the new workflow they demanded.

### Deployment and maintenance: scaling up or closing down

One year after the pilot, only two out of the five interventions had been scaled up and put into use by all five hospitals, thirty-three healthcare centres and fourteen community services. These were the attached-file ADL and the web calendar. A routine for how to use the follow-up form and the surveillance list was documented but not communicated to or known by the users. After the pilot evaluation, senior management made a policy decision not to implement videoconferencing as an alternative way of conducting care meetings. This meant it was up to each care unit to decide whether they would implement it or not, resulting in none using it.

Five years after the pilot, the ADL form was still in use. However, the IT system had been replaced with a new one that contained a structured form for the ADL. The new IT system does not report statistics on the frequency of use of the ADL, but a manual spot check indicated that the form was used regularly. Senior management had decided to decommission the web calendar, and as the new IT system did not offer any similar function, a high priority was given to introducing said function by the end users in the backlog. Follow-up documentation and a surveillance list were available under the new IT-system and were implemented at all units. Regulations on how to use these to meet long-term patient care needs after discharge was established between the county and the communities four years after the pilot. Even so, the adoption rate was low: the statistics showed that out of 1900 established discharge plans, only thirty-two percent were followed up on after discharge. At the beginning of year five, senior management had also made a policy that videoconferencing was to be implemented as an alternative way to perform DPC meetings. The result shows that 1444 meetings were performed via video eight months after implementation, which was nearly eighty percent of the DPC meetings conducted at that time.

## Discussion

The aim of this study was to explore the experiences of nursing staff with an agile development process from initial requirements to the deployment of its outcomes of ICT solutions aimed at supporting patient-discharge planning. The qualitative content analysis of focus group interviews revealed one theme, *it’s all about time and timing*, and four categories, *allot time for time, shared time saves time, gaps in timeliness hinder time and timing,* and *not if, but when and how.* The findings show that the personnel valued the agile development process and the possibility of being involved in and influencing the development of the new ICT solutions. They also perceived that the developed ICT solutions supported the DP process. However, both advantages and disadvantages need to be considered during the development and use of new ICTs. In this study, the importance of time and timing throughout the development process became very clear, and factors that could be a help or hindrance to time and timing were revealed.

### Starting points

The starting points of the initiative included the application for funding and the project’s launch. This work engaged senior and middle management representatives at the regional and municipality levels, along with the project manager from the university. With their oversight, the budget, the available resources, and an overall timeframe and road map were established. Middle management recruited driven, interested RNs, DNs and HCOs from amongst their nursing staffs to participate in the project. A scrum master from the university and the technological project leaders from the region and from two external companies were chosen. The performance of this stage of the project differs from that of a waterfall methodology even though some of the tasks themselves are pretty much the same, e.g., application, budget, resources and a timeframe. The role of scrum master is, however, specific to an agile methodology [40]. The scrum master acts as a coach for the team by lending support in the development process. On the other hand, in a waterfall methodology’s the project manager is the leader and decision-maker responsible for the deliverable by managing the scope, budget, time and resources efficiently. In this project, both roles were appointed and clearly defined. This positively affected the development process since the scrum master secured time and timing for the support and guidance of the team, while the project manager secured the logistical aspects of the project. On the other hand, the product’s owner was not clearly defined or communicated. In this case, senior management at three care providers (the region and two municipalities) should have been viewed as product owners. Even so, a great deal of communication occurred between the project manager and the region’s senior management. To facilitate collaboration when more than one organisation/company is the product owner, a steering group is recommended [40]. An agile steering group’s work is to actively follow the development process with an explorative attitude towards the project’s result. They usher the work towards the goal without governing the result, whereas a steering group in a waterfall project directs the work to achieve the expected result. The lack of a well-defined agile product owner can be interpreted as affecting the development process in a negative way in this case as directions did not occur in a timely fashion due to unclear communication. This would probably have been the case even if a waterfall methodology had been used. Both agile and waterfall methodologies require a communication plan to be established early on in the process [41] in addition to defining roles, even if they differ both in terms and mission [42]. Timely information is crucial [43]. If it is communicated too early, it might be forgotten; if it comes too late, one runs the risk of delays in the process. Timely information gives the recipient time to absorb and process the information necessary to complete the work as planned.

### Iteration

In this stage, workshops and meetings were conducted to map the discharge planning process and its sub-processes in greater detail to identify activities, actors, actual problems, needs and stakeholders. A product vision and a detailed timeframe/road map were established. Requirements were prioritized and documented, and premature solutions were designed, developed, and tested before the next sprint started it all over again. This work was done in close collaboration between the scrum master, the nursing staff and the development team and also among the project manager, scrum master and all project leaders. Senior and middle management were not involved in this stage. In an agile project, the product owner/customer should communicate with the project team on a daily basis, which can be a great difficulty [43]. The project requires an active customer, and if customer commitment waning runs, a high risk exists of project failure [44]. RNs, DNs and HCOs expressed frustration about middle management making decisions to cooperate in the development project without giving them the tools to do so. This demonstrates that engagement from management is essential to successfully adopting new practices or products for daily use [45]. Healthcare systems are complex in their design. Their advanced, interacting networks make change very challenging to implement and sustain. Hofflander et al. [46] suggest that managers at all levels should receive more information and training about how to encourage staff to become involved in designing their everyday work and in the implementation process. They found that ongoing organizational support is necessary for effective leadership throughout the start-up, development, implementation, and evaluation stages. Urquhart et al. [18] found that it can be difficult for middle managers to support the staff since they had other roles and responsibilities that required much of their time, thus limiting their decision-making powers. At the same time, managers perceived themselves to be planners, coordinators, facilitators, motivators, and evaluators during the development and implementation of new tools and practices. However, they often felt inadequately prepared for and lacking in formal knowledge or training regarding these roles.

In agile projects, customer involvement and feedback are closely integrated into the development process, resulting in better prioritization of functions and higher customer value. Thus, the chance of reaching a successful adoption of new interventions is increased by using an agile process. Nurses are already familiar with agile methodologies through their collaborative and iterative methods of providing care [47]. They adapt plans of care based on patient needs and resources amidst frequent staffing constraints. Every day they develop new techniques based on the current literature and their training and experience and then apply these techniques to a given situation. Nurses are therefore well suited to be involved in ICT development and implementation, as they can use these well-developed agile techniques in the process. The participating RNs, DNs and HCOs described themselves as driven and willing to invest time in the development process; however, they also emphasized the necessity of middle management, colleagues, and themselves quickly allotting time for involvement in the development phase and practice with the tool. They stated that this was essential for project participation, especially in the iteration stage. Both agile and waterfall methodologies need “CRACK” performers to reach success; that is, participants who are collaborative, representative, authorized, committed and knowledgeable [44]. Waterfall methodologies, however, do not require customer participation full time in all stages as agile methodologies do. Even though agile development processes are performed over a limited period of time, it is still time-consuming from the point of view of the customer. While the RNs, DNs and HCOs in our study were fully aware of the time involved in the development process, they still thought it was preferrable for future development work. Again, the question is not *if* the personnel should be involved and engaged in the process, but rather *when* and *how*. The COVID-19 pandemic has required compromises and creativity from the healthcare system to meet the new demands [48]. Agile project management methodologies have been implemented where clinicians have been needed in order to lead the rapid and effective development and implementation of new clinical standards, ethics, analytics, education and communication.

This study found that nursing staff sharing their schedules saves time overall and that transparency facilitates collaboration, planning and coordination by increasing providers’ understanding of each other’s roles and responsibilities. In waterfall methodologies, the development process is linear without room for revision, and the end users only becoming involved in defining requirements and in the test period when the ICT is already constructed. This is less time-consuming for the end users. Even though, this approach to an early co-design phase may get the technology to a reasonably mature stage relatively quickly, an iterative and adaptive co-design of the technology increases the chance of it becoming sustainably embedded in daily practice [49].

### The pilot

In the pilot phase, training on the developed ICT solutions was achieved for all staff at the participating units. It was followed by a twelve-month test period after the last sprint was launched. The agile development process increased the participants’ readiness for change by offering them time to learn, practice, engage with, reflect on and thereby adopt the ICT as an aid to daily practice. Balje, Carter, Velthuijsen [50] argue that an agile development process, with its user involvement and product visibility, can be used as a change management approach in healthcare innovation projects to reach user acceptance and innovation adoption. Change management can be seen as the process of supporting individuals and organizations in the transition from an old method to a new way of doing things [51]. Change can either be planned or emergent [52]. Planned change is described as a top-down approach with a clearly defined goal, whereas emergent change is an unanticipated bottom-up approach. Some commentators consider the combination of both to be the preferred method of achieving organisational change [53]. Kotter [54] developed a generic plan to facilitate emergent change. This change management model consists of three phases: creating a climate for change, engaging and enabling the entire organisation, and implementing and sustaining the change. In total, eight steps are involved. It has been shown that the agile methodology can support organisations through these phases and thereby support the adoption of new tools and practices [50]. However, the main risk is that the agile methodology is limited to the development phase, therefore leading to a loss of implementing and sustaining the change due to a long lag time lag between the development, pilot and scaling-up phases. In this study the nursing staff noted that the time between the training and the pilot was too long. Many of them perceived that they had forgotten what they had learned before they could try it, resulting in a time and performance pressure that had to be overcome before they could practice their skills. They also emphasized the importance of receiving suitable support in a timely manner during the pilot phase. It is important to offer the staff training exactly when needed with a supervisor or instructor available at the workplace so that user problems or data entry errors can be prevented [47].

### Deployment and maintenance: scaling-up or closing down

In this phase, the pilot was evaluated, and the results were delivered to senior management so they could make a decision for the intervention’s future. The project was then ended, and the deployment and maintenance of the ICT solutions was studied after one year and five years.

Even though representatives from all three healthcare professions described the videoconferences as providing economic benefits and environmental savings—in addition to the time savings from DNs not having to travel to the DPC, senior management decided not to scale it up after the pilot. The second product developed—the transparent and shared electronic calendar—was also perceived to be of value and assisted with the timing and coordination of personal resources across organizational boundaries for successful DPCs. It was initially implemented and frequently used by the RNs, DNs and HCOs, but when the new IT system was introduced, the calendar too was abolished by senior management. This can be interpreted as the nursing staff, through their engagement, collaboration, training and skills, recognising the tools’ value for themselves, their patients and the patients’ relatives. Senior management, on the other hand, had not been engaged through the development process and were not prepared to implement change. There is a significant relationship between the support of senior management and their commitment to the success of agile projects [55]. Communication is essential to involvement, and in agile projects, direct communication and feedback play a vital role in linking the technology team, the project manager/scrum master, the team members and the management. Also, ill-defined project scopes, requirements and planning, along with ill-defined roles and lack of management competence, have been identified as risk factors [56]. The low level of commitment from senior management was probably due to a combination of unclear project ownership, ill-defined roles and lack agile competence among management, which led to them not being prepared to implement change.

The third, fourth and fifth products resulting from the agile development plan were the attached ADL file, the follow-up form and the surveillance list. These were perceived as being supportive by the nursing staff, but they also generated excessive work for them due to double documentation across different IT systems. These documents were not adopted due to the lack of new routines for how the work should be done. Changing health-professional behaviour, methods of treatment and healthcare-system structure has proven to be a hindrance to the implementation of new ITC systems [57]. A change in the work process may therefore take a long time to implement. Compared to systems in other industries, health professionals are less inclined to experiment with new solutions because they affect their patients’ life and health [58]. Resistance to change can also arise from experience with previous implementations that were abandoned, perceived as tedious rather than innovative, or met with initiative exhaustion [59]. Therefore, it is even more important that the organisation management show full support and acceptance of the agile methodology so that they do not become a barrier to transition [60]. They need to understand that successful changes are about shifts in mindsets, not just processes. Agile methodologies require a decentralized management style that works more from the bottom-up, as opposed to the waterfall methodologies that uses a top-down approach. The highest management levels need to relinquish micromanagement and be open to new processes that use scope, time and cost to create a new overarching system resulting in better quality and increased business value. At the same time, they need to empower and encourage project teams to make decisions. An important reminder is that the agile methodology is not limited to technology and innovation projects; it is also valuable for clinical-care redesign [61]. The agile methodology could be used by teams to rethink their care model, starting and working closely with patients, their relatives and the larger community to ensure a care experience tailored to their goals and needs. The agile approach provides means to explore, integrate and adapt new and emerging scientific knowledge and tools, as well as the chance to respond to changing patient and family needs and expectations to create a truly adaptive, responsive caring system. The agile approach can be used by innovative healthcare organizations to deliver patient-centred care in a better way than the waterfall methodology can.

#### Limitations

The low response rate to the evaluation form can be seen as a weakness of this study. The low response rate might have affected the results [37] and perceived problems or difficulties with the videoconferencing might not have been reported. However, the participants described similar problems in both the evaluation form and the FGs. The sizes of the FGs were smaller than what Kruger and Casey [36] suggest. The small groups were not a problem in this study because the participants were loquacious and expressive and reported a variety of aspects and reasoning within each group. There is a potential risk that the participants of FGs will affect each other and strive for consensus. However, the participants in each FG were more or less familiar with each other through their daily collaboration on DPs and all participants contributed to the results. Credibility was the goal during data collection as the authors avoided asking leading questions or finishing participants’ statements. During the analysis, close attention was paid to the text until the final stage when the focus shifted to interpreting and formulating a theme. Alternative interpretations were discussed amongst the authors. All authors took part in discussions during each step of the study, which strengthened the trustworthiness of the results. This was mainly a qualitative study. While the results cannot be generalized, they can be transferred to similar situations and contexts.

## Conclusion

This study on developing and testing ICT solutions points out the importance of time and timing. The findings show a need for time to prepare, understand and adopt new systems. Additionally, the need for timing the preparation, understanding and adoption of the ICT solution to best meet the needs of individuals, collectives and organizations, and sometimes also among different collectives and organizations, was shown to be crucial for the smooth and successful adoption of a new ICT solution. This study showed that the agile-inspired development process offered the end users involvement in the development process, which gave them time to adjust to change, both individually and collectively. It is a huge challenge to implement agile methodologies in healthcare organizations since they are traditionally governed by a top-down hierarchy. However, the agile methodology does not offer organizational changes. Rather, it requires close collaboration between participants in the development project and management to achieve fast, well-timed organizational change. The time or timing failing in the development or implementation process results in a huge risk of non-adoption of the new practice or tools on different organizational levels.

## Data Availability

The quantitative datasets generated by the IT system and the qualitative data from the focus groups that were analysed during the current study are not publicly available due to the terms of Swedish confidentiality policies but are available from the Department of Research and Education, Region Norrbotten, upon reasonable request.
